# Retrospective cohort study of Advanced HIV disease among children and adolescents in Uganda: Characteristics, clinical outcomes, and rate of mortality

**DOI:** 10.1371/journal.pone.0338360

**Published:** 2026-07-02

**Authors:** Calvin Epidu, Rogers N. Ssebunya, Freddrick E. Makumbi, Edgar Sserunkuma, Emmanuel Tumwine, Patrick Kizza, Michael Juma, Henry Balwa, Betty Nsangi, Albert K. Maganda, Denise J. Birungi, Arthur G. Fitzmaurice, Dithan Kiragga

**Affiliations:** 1 Baylor College of Medicine Children’s Foundation, Kampala, Uganda; 2 Department of Epidemiology and Biostatistics, School of Public Health, Makerere University, Kampala, Uganda; 3 Division of Global HIV & TB, Global Health Center, United States Centers for Disease Control and Prevention, Kampala, Uganda; Nigerian Institute of Medical Research, NIGERIA

## Abstract

**Background:**

People diagnosed with advanced HIV disease (AHD) are at high risk for mortality even after starting antiretroviral therapy (ART). We determined characteristics, clinical outcomes, and risks of mortality among children and adolescents diagnosed with AHD in western Uganda.

**Methods:**

We conducted a retrospective cohort analysis of routinely collected program data of children and adolescents living with HIV (CALHIV) aged 0–19 years, from outpatient HIV clinic electronic medical records in 48 high-volume health facilities in two regions of western Uganda (Fort Portal and Hoima). Data for clients who initiated ART during January 2016—July 2023 were analysed. AHD was defined as a CD4 cell count <200 cells/μL, or WHO stage 3 or 4, or any child younger than 5 years of age living with HIV who had been on ART for more than 12 months and virally non-suppressed (≥1,000 copies). We used descriptive statistics (i.e., frequencies and percentages) to summarise characteristics and treatment outcomes. Kaplan-Meier curves were used to estimate survival overall and by clients’ characteristics; log-rank tests were used to compare survival functions. A gamma-shared frailty model was used to determine factors associated with the rate of mortality. Effect measures were summarized using adjusted hazard ratios (aHRs) with corresponding 95% confidence intervals (95%CI).

**Results:**

A total of 5,143 CALHIV, including 3,067 (59.6%) females, with a median (interquartile range [IQR]) age of 10 (9) years were assessed. Overall, AHD was high (18.1%) and varied by age—0–4 years (68.4%), 5–9 years (12.6%), 10–14 years (13.2%), and lowest among adolescents, 15–19 years (7.7%). Just over half of the CALHIV with AHD were active in care (51.5% [480/932]), about a quarter (26.4% [264/932]) had transferred out, 13.8% (129/932) were lost to follow-up, and 8.3% (77/932) had died. Survival was significantly higher in CALHIV who were not malnourished compared to those with malnutrition (p = 0.001). Overall mortality rate per 100 person-years among CALHIV with AHD was 4.1 (95%CI:3.2–5.2) and was significantly higher among those who had been on ART for 3 months or less (27.3; 95%CI: 20.6–36.2) compared to 6 months or more (1.0; 95%CI: 0.6–1.7).

**Conclusion:**

Advanced HIV Disease among CALHIV in western Uganda was consistent with what has been published elsewhere. Risk of death differed by nutrition status and was high among those on ART three months or less. Early screening and management of malnutrition, as well as early ART initiation and adherence initiatives, might improve outcomes and reduce AHD-related mortality among CALHIV.

## Introduction

Amidst the global success in scaling up antiretroviral therapy (ART) coverage, children and adolescents living with HIV (CALHIV) remain hidden and left behind compared to adults. In 2023, nearly four in ten infants with HIV missed out on a timely diagnosis [1]. Each day in 2024, approximately 712 children aged 0–19 years became infected with HIV, and approximately 250 died from AIDS-related causes, mostly due to inadequate access to HIV prevention, care and treatment services [2]. The United Nations Children’s Fund (UNICEF) estimated close to 760,000—about 55% of the 1.38 million children aged 0–14 years living with HIV—were not on ART in 2024 [3].

Treatment outcomes among children and adolescents 0–19 years old have improved since before the test-and-treat era but remain suboptimal. Retention-in-care rates, for example, improved globally after implementation of the 2017 guidelines to 55% in children aged 2–4 years, 65% in children aged 5–14 years, and 55% in children and adolescents aged 15–19 years [4]. Continuity in HIV care among CALHIV has also been suboptimal, with younger (<5 years) compared to older (5–9 years) pediatric patients being twice as likely to die within 90 days after initiation of ART and older adolescents (15–19 years) demonstrating greater non‐engagement in care in a large cohort in Tanzania [5]. Viral load suppression rates have also been shown to be lower compared to adults 18 years and older (i.e., 36% vs. 44% one year after ART initiation, 30% vs. 36% after year 2, and 24% vs. 29% after year 3) [6].

The World Health Organization (WHO) defines Advanced HIV Disease (AHD) as CD4 cell count <200 cells/mm^3^, WHO stage 3 or 4, or any child younger than 5 years of age living with HIV who has been on ART for more than 12 months and has an unsuppressed HIV viral load (>1,000 copies/ml) [7]. AHD is associated with poor treatment outcomes among people living with HIV (PLHIV), including increased risk of opportunistic infections and death [8] as well as lower health-related quality of life (i.e., a worse physical or mental health status) [9]. According to the 2023 policy brief of the WHO [8], one fifth of the admitted AHD cases did not survive their hospital admission, and of those who survived, nearly a third died or were readmitted to the hospital within a year. “The path that ends AIDS: 2023 UNAIDS Global AIDS Update” report posited that AHD had become an increasing issue partly due to changing profiles of people with AHD including those who interrupt treatment and return to care [10]. The WHO recommends screening, treatment, and prophylaxis for major opportunistic infections; initiation of ART within 7 days of diagnosis; and intensified adherence support as key interventions to reduce morbidity and mortality due to AHD.

Available evidence on the burden of AHD is mostly among adults, with a paucity of data on treatment outcomes including risk of mortality among CALHIV aged 0–19 years. Although testing and treatment for all PLHIV in Uganda started in 2016 [11], approximately 30% newly identified PLHIV (all ages) present to care with CD4 cell counts < 200cells/mm3 and approximately 25% return to care with AHD after treatment interruption [12]. As highlighted in the WHO guidance [7], Uganda’s treatment guidelines note that clinically stable children who have received 12 months of ART should not be considered to have advanced HIV disease. Our study, therefore, set out to determine characteristics, treatment outcomes, and rate of mortality following AHD diagnosis among CALHIV in mid-western Uganda.

## Materials and methods

### Study design, setting, and population

We conducted a retrospective cohort analysis of data of CALHIV (0–19 years) in HIV care in Hoima and Fort Portal regions in western Uganda. Data were abstracted from out-patient HIV clinic electronic medical records (EMRs) in 48 health facilities supported by the U.S. President’s Emergency Plan for AIDS Relief (PEPFAR) through the U.S. Centers for Disease Control and Prevention (CDC). Probability proportionate to sample (PPS) approach was used to identify the 48 facilities. These facilities with EMR contributed 80% of the active CALHIV during the April-June 2023 quarter and included primary (Health Centre III) and secondary (district hospitals) level facilities. We included data within the EMR from the 48 health facilities for CALHIV (0–19 years) who started ART during January 2016—July 2023.

Clients included in this evaluation were managed per Uganda’s HIV care and treatment guidelines [12]. All facilities included in this study provided the basic required HIV services including baseline CD4 count testing and screening and diagnosis of cryptococcal meningitis and tuberculosis (TB). The analysis covers the test-and-treat period for all PLHIV operationalized in these regions beginning January 2016. Clients with missing date for initiation of ART but with date confirmed HIV-positive were assigned the HIV-positive test date. We excluded observations with CD4 count values evaluated before January 2016.

### Data collection procedures

With support from facility-based medical records assistants (MRAs), regional monitoring and evaluation officers, and health information system (HIS) officers, routinely collected data were abstracted from EMRs in the health facilities within the two regions of Hoima and Fort Portal. Data were abstracted on 30 April 2024 and were fully anonymized before being made available to the research team. The key data variables abstracted included client sociodemographic characteristics, HIV diagnosis, baseline CD4 count, WHO clinical staging, TB status, cryptococcal meningitis status, and last clinic encounter date. The abstracted data covered five clinic visits. Data quality checks were conducted, and missing data were cross-referenced from registers and cleaned accordingly. Data were cleaned using Microsoft Excel and exported to STATA statistical software version 18.0 for analysis.

### Variable measurements

The main outcome of interest was AHD, defined as CD4 cell count <200cells/mm3 or WHO stage 3 or 4 in adults and adolescents, and all children under 5 years of age regardless of CD4 count [12]. However, children younger than 5 years old who had been on ART for more than one year and were virally suppressed were not classified as AHD. We determined both AHD at initiation of ART based on baseline CD4 count values and across the study period for each of the study participants based on baseline CD4 count, WHO clinical stages, and viral load suppression values on each of the subsequent clinic visits. Secondary outcomes of interest included active in care, died, transferred out (TO), and lost to follow-up (LTFU). CALHIV who reached 28 days after their scheduled clinic appointment date without returning to care were considered LTFU. All TO clients had a documented outcome as “Transferred out” in the EMR by the time of data abstraction. CALHIV were categorised as active if their last clinic encounter was within 28 days or their next clinic visit date was ahead of the date of data abstraction.

Time-to-death was estimated from time of initiation of ART to documented date of death, while administrative censoring was considered at month 60 if no event occurred before the 60-month follow-up. Other variables of interest included nutrition status and any confirmed stage 3 or 4 condition (TB and cryptococcal meningitis) or a classification of WHO clinical stage 3 or 4 at any of the last five clinic visits. The last clinic visit date was considered as date of transfer for clients documented as TO but with missing date. For CALHIV below and above 60 months, nutrition status was based on available mid-upper arm circumference (MUAC) measurements routinely done for CALHIV in HIV clinics (i.e., color code “Red” for severe acute malnutrition, “Yellow” for moderate acute malnutrition, and “Green” for no malnutrition). For CALHIV with missing MUAC values (below and above 60 months), we used their weight for age z-scores to categorise their nutrition status. Despite their use being limited to children younger than 60 months of age, in the national guidelines [13], MUAC tapes can be used to assess and classify nutrition status of clients both below and above 60 months of age.

### Statistical analysis

We conducted an exploratory analysis with descriptive statistics on all key variables of interest. Chi-square tests were used to assess associations between AHD and clients’ sociodemographic and health facility characteristics. The rate of death was determined as the number of CALHIV who died during the follow-up study period divided by person-time-at-risk and reported per 100 person-years at risk (pya). We right censored the data after five years of follow-up. Kaplan-Meier method estimation was used to obtain the survival probabilities. Kaplan-Meier curves were used to estimate survival and by other clients’ characteristics, and the significance of the differences was assessed using log-rank tests. P-values of <0.05 were considered statistically significant. To determine independent factors associated with time-to-death, we fitted a proportional hazard model with a gamma shared frailty. Effect measures were summarized using adjusted hazard ratios and their 95% confidence intervals (95%CI).

### Ethics

This activity was reviewed by U.S. CDC and was determined to be non-research; it was conducted consistent with applicable federal law and CDC policy, 45 C.F.R. part 46, 21 C.F.R. part 56; 42 U.S.C. §241(d); 5 U.S.C. §552a; 44 U.S.C. §3501 et seq. An exemption from an Institutional Review Board (IRB) was granted by the Makerere University School of Public Health Higher Degrees, Research and Ethics Committee in Uganda. Consent for study participants was not required in this study because it was based on routinely captured secondary data in health facilities.

## Results

### Characteristics of study participants

Table 1 shows both sociodemographic and clinical characteristics of study participants. High proportions of CALHIV were from lower-level health facilities (Health Centre III (HCIII); 50.2% were categorised as WHO clinical stage 1 or 2 (93.7%) and were initiated on either non-nucleoside reverse transcriptase inhibitors (NNRTIs) (41%) or integrase inhibitors (37.1%) as their baseline ART regimens. Of those with documented viral load values, most (81.7%, 2,741/3,354) were virally suppressed. The proportion of CALHIV with history of TB diagnosis in this study population was 2.3% (117/5,143).

**Table 1:** Characteristics of CALHIV assessed for AHD between January 2016 – July 2023 in two regions of Uganda.

Characteristics		Frequency	Percent (%)
	Overall	N = 5,143	
**Sex**	Male	2,076	40.4
	Female	3,067	59.6
**Age group (years)**	0 - 4	633	12.3
	5 - 9	1,680	32.7
	10 - 14	1,262	24.5
	15 - 19	1,568	30.5
**Region**	Hoima	3,045	59.2
	Fort Portal	2,098	40.8
**Health facility level**	HCIII	2,581	50.2
	HCIV	1,405	27.3
	Hospital	1,157	22.5
**Baseline CD4 count**	< 200 cells/μl	241	4.7
	≥ 200 cells/μl	1,168	22.7
	Missing	3,734	72.6
**WHO clinical stage**	Stages 1 & 2	4,668	90.7
	Stages 3 & 4	382	7.4
	Missing	93	1.8
**ART regimen** ^**β**^	NNRTIs	2,109	41.0
	Protease inhibitors	977	19.0
	Integrase inhibitors	1,906	37.1
	Others^¶^	151	2.9
**Viral load (most recent)**	<1,000 copies/ml	2,741	53.3
	>=1,000 copies/ml	613	11.9
	Missing	1,789	34.8
**Median duration on ART**	18 months (iqr = 50)		
**History of TB diagnosis**	Yes	117	2.3
	No	5,026	97.7

**β** – Baseline ART regimen, **¶** - Other first line regimens, **iqr** – Interquartile range, **HC** – Health Centre, **CD4** – Cluster of Differentiation 4, **ART** – Antiretroviral therapy, **NNRTIs** – Nucleoside reverse transcriptase inhibitors, **TB** – Tuberculosis

### Advanced HIV Disease and treatment outcomes by individual characteristics

AHD at initiation based on CALHIV with available baseline CD4 count values was 17.1% [15.1–19.1], (241/1409). It varied by age group: 0–4 years (16.1%), 5–9 years (25.1%), 10–14 years (21%), and 15–19 years (10.3%). Twenty-one (13/61) and twenty-five (34/139) percent of CALHIV with MAM and SAM, respectively, had AHD. Table 2 highlights the proportion of CALHIV with AHD across the study period, overall and stratified by client characteristics. Eighteen percent of CALHIV had AHD, 18.1% [17.1–19.2], (932/5,143) and with higher proportions among children 0–4 years (68.4%) [64.6–72.0]. Advanced HIV did not differ by region (Hoima at 18.5% (563/3,045) and Fort Portal at 17.6% (369/2,098)). More than half (61.4%) with AHD had been on ART for 12 months or less. Where data on nutrition status were available, AHD was common among CALHIV clients with moderate, 24.5% (50/204), or severe acute malnutrition, 29.1% (175/601).

**Table 2:** Advanced HIV Disease (AHD) among CALHIV assessed between January 2016 – July 2023 in Hoima and Fortportal regions, Uganda.

		AHD^µ^ (n/N)	AHD (%)	p-value
**Characteristics**	Overall	932/5,143	18.1	
**Sex**	Female	510/3,067	16.6	0.001
	Male	422/2,076	20.3	
**Age group (years)**	0 - 4	433/633	68.4	0.001
	5 - 9	212/1,468	14.4	
	10 - 14	167/1,262	13.2	
	15 - 19	120/1,568	7.7	
**Region**	Hoima	563/3,045	18.5	0.205
	Fort portal	369/2,098	17.6	
**Duration on ART** ^**ρ**^	0 - 6	383/1,582	24.2	0.001
	7 - 12	189/642	29.4	
	13 - 24	90/630	14.3	
	>24*	270/2,289	11.8	
**Nutrition status**	No Malnutrition	267/1,620	16.5	0.001
	Moderate Acute Malnutrition	50/204	24.5	
	Severe Acute Malnutrition	175/601	29.1	
	Missing	440/2,718	16.2	
**ART regimen** ^**α**^	NNRTIs	113/677	16.7	0.033
	Protease Inhibitors	112/499	22.4	
	Integrase Inhibitors	698/3,896	17.9	
	Others^¶^	9/71	12.7	

**µ –** AHD across the study period for each of the study participants, **ρ** – Duration on months, **α** – Most recent clinic visit regimen, **ART** – Antiretroviral therapy, **NNRTIs** – Nucleoside reverse transcriptase inhibitors, **¶** - Second line containing both integrase and protease inhibitors. *170/270 (63%) CALHIV were aged 5–14 years, 40/270 (15%) were 15–19 years.

Table 3 highlights HIV treatment outcomes among those with AHD. Overall, half (51.5%, 480/932) were active in care, 8.3% (77/932) had died, 13.8% (129/932) were LTFU, and 26.4% (246/932) had been TO from their primary HIV care facilities. Deaths were more common among CALHIV clients with AHD (8.3%, 77/932) compared to non-AHD (2.8%, 118/4,211). The proportion of CALHIV who died varied by age group: 8.3% (36/433)] among 0–4 years, 10.4% (22/212) among 5–9 years, 6.0% (10/167) among 10–14 years, and 7.5% (9/120) among those aged 15–19 years. The proportion of CALHIV with AHD who died was higher in Hoima Region (9.4%) compared to Fort Portal Region (6.5%) but was not statistically significantly different (p = 0.115). Among CALHIV who had been on ART for 6 months or less, a significantly higher proportion (15.9%, 61/383) died compared to those who had been on ART much longer (i.e., 4.8% [7–12 months], 2.2% [13–24 months], and 1.9% [>24 months]). A similarly high proportion (20.6%, 36/175) of CALHIV with AHD who had died also had severe acute malnutrition. The proportion of CALHIV who died was statistically significantly higher among CALHIV with AHD (8.3%, [77/932]) compared to those without AHD (2.8% [118/ 4,211]).

**Table 3:** Treatment outcomes among CALHIV with Advanced HIV Disease between January 2016 – July 2023 in Uganda.

Variable			Total (N)	Active (n, %)	Died (n, %)	LTFU (n, %)	Transferred out (n, %)
**Overall**			932	480 (51.5)	77 (8.3)	129 (13.8)	246 (26.4)
**Sex**	Male	422		217 (51.4)	43 (10.2)	60 (14.2)	102 (24.2)
	Female	510		263 (51.6)	34 (6.7)	69 (13.5)	144 (28.2)
**Age group (years)**	0–4	433		250 (57.7)	36 (8.3)	47 (10.9)	100 (23.1)
	5–9	212		100 (47.2)	22 (10.4)	34 (16.0)	56 (26.4)
	10–14	167		74 (44.3)	10 (6.0)	26 (15.6)	57 (34.1)
	15–19	120		56 (46.7)	9 (7.5)	22 (18.3)	33 (27.5)
**Region**	Hoima	563		293 (52.0)	53 (9.4)	81 (14.4)	136 (24.2)
	Fort Portal	369		187 (50.7)	24 (6.5)	48 (13.0)	110 (29.8)
**Duration on ART** ^**ρ**^	0–6	383		107 (27.9)	61 (15.9)	68 (17.8)	147 (38.4)
	7–12	189		124 (65.6)	9 (4.8)	18 (9.5)	38 (20.1)
	13–24	90		51 (56.7)	2 (2.2)	13 (14.4)	24 (26.7)
	>24	270		198 (73.3)	5 (1.9)	30 (11.1)	37 (13.7)
**Nutrition status**	No Malnutrition	267		178 (66.7)	8 (3.0)	29 (10.9)	52 (19.5)
	Moderate Acute Malnutrition	50		31 (62.0)	2 (4.0)	4 (8.0)	13 (26.0)
	Severe Acute Malnutrition	175		55 (31.4)	36 (20.6)	38 (21.7)	46 (26.3)
	Missing	440		216 (49.1)	31 (7.1)	58 (13.2)	135 (30.7)

**ρ** – Duration on months, **ART** – Antiretroviral therapy, **LTFU** – Lost to follow-up.

### Survival experiences by age group and nutrition status among CALHIV with AHD

**Fig 1:**
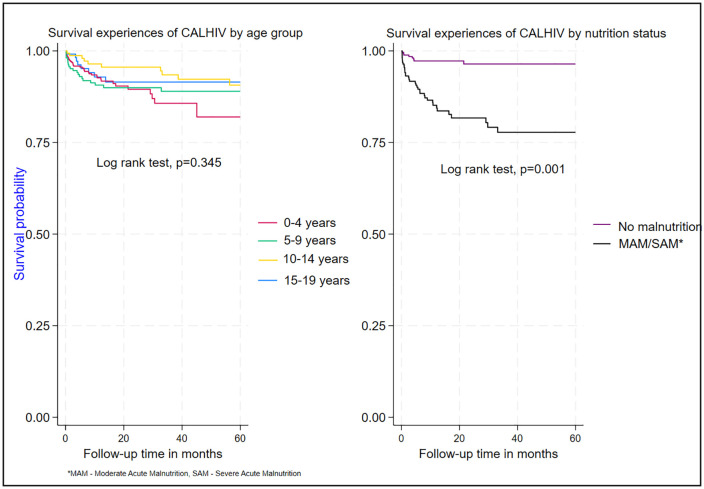
Survival by age group and nutrition status among CALHIV with Advanced HIV Disease between January 2016 – July 2023 in Uganda.

Fig. 1 is the KM curve highlighting the probability of survival of CALHIV with AHD. The overall probability of survival was above 75% and varied by age group and nutrition status. Children 0–4 years old and 5–9 years old had lower survival experience compared to those 10 years and older, although they were not statistically significant (p-value = 0.345). CALHIV with severe or moderate acute malnutrition had significantly lower survival rates compared to those without malnutrition (p-value = 0.001). Generally, irrespective of age and nutritional status, death occurrences were highest within the first 15 months of starting ART and then plateaued. Deaths occurred even after 24 months of starting ART across all age groups and among those with malnutrition.

### Mortality rate and associated factors of mortality among CALHIV with AHD

Compared to CALHIV without AHD, mortality rate was significantly higher among those with AHD, i.e., 4.1 (3.3–5.2) versus 0.9 (0.8–1.1) per 100 pya. Mortality rate among CALHIV with AHD as shown in Table 4 was predominantly seen among those who had been on ART for 3 months or shorter (27.3 [95%CI: 20.6–36.2] per 100 pya,), followed by CALHIV who had spent 4–6 months on ART (9.8 [95%CI: 5.4–17.6] per 100 pya). Males had a higher mortality rate compared to females (5.2 [95%CI: 3.9–7.0] vs. 3.3 [95%CI: 2.3–4.6] per 100 pya). The rate of death in children 0–4 years old was 6.5 (95%CI: 4.7–8.9) per 100 pya, 3.9 (95%CI: 2.5–6.1) per 100 pya among 5–9-year-olds, and 2.9 (95%CI: 1.5–5.8) per 100 pya among 15–19-year-olds. CALHIV with moderate and severe acute malnutrition were more likely to die (adj.HR = 8.8; 95%CI: 2.7–28.3) compared to those who were not malnourished. CALHIV who had been on ART for 3 months or less had higher risk of death compared to those on ART for 6 months and longer (adj.HR = 205.2; 95%CI: 43.1–977.3).

**Table 4:** Mortality rate and associated factors of mortality among CALHIV with Advanced HIV Disease between January 2016 – July 2023 in Uganda.

Variable	Total observations	Number of events^π^	Person years per 100	Mortality rate	95% CI	Unadj.HR	95% CI	Adj.HR	95% CI
**Overall**	932	74	17.92	4.13	3.29 - 5.19				
**Sex**									
Female	510	32	9.85	3.25	2.30 - 4.59	**1.0 (ref)**		**1.0 (ref)**	
Male	422	42	8.07	5.20	3.85 - 7.04	1.63	1.03 - 2.58	1.82	0.68 - 4.88
**Age group (years)**									
15 - 19	120	8	2.74	2.92	1.46 - 5.84	**1.0 (ref)**		**1.0 (ref)**	
10 - 14	167	10	4.53	2.21	1.19 - 4.10	0.83	0.33 - 2.10	0.25	0.02 - 2.76
5 - 9	212	20	5.10	3.92	2.53 - 6.08	1.38	0.61 - 3.13	0.43	0.06 - 3.37
0 - 4	433	36	5.55	6.48	4.68 - 8.99	1.49	0.69 - 3.23	0.55	0.08 - 3.77
Duration on ART*									
> 6	549	15	15.03	0.99	0.60 - 1.66	**1.0 (ref)**		**1.0 (ref)**	
4 - 6	124	11	1.13	9.75	5.40 - 17.61	82.26	15.25 - 443.81	**17.5**	**3.75 - 81.22**
0 - 3	259	48	1.76	27.28	20.56 - 36.20	5364	731.7 - 39324	**205.2**	**43.1 - 977.3**
**Nutrition status** ^**¥**^									
No Malnutrition	267	8	5.66	1.41	0.71 - 2.83	**1.0 (ref)**		**1.0 (ref)**	
MAM or SAM	225	36	3.90	9.23	6.66 - 12.80	6.13	2.85 - 13.20	**8.78**	**2.72 - 28.34**

**¥** - based on complete case analysis, 440 cases had missing data on nutrition status, *Duration in months, **MAM** – Moderate Acute Malnutrition, **SAM** – Severe Acute Malnutrition, **Unadj.HR** – Unadjusted hazard ratio, **Adj.HR** – adjusted hazard ratio. **π** – Three events (deaths) occurred after the 5 years of follow-up.

## Discussion

Our study showed that 18 percent (18.1%) of CALHIV across the study follow-up period had AHD, with children younger than 5 years old, those who had been on ART for ≤6 months, and those with malnutrition significantly associated with AHD. AHD at ART initiation was 17.1% (241/1409). Overall mortality risk among those with AHD was higher than among those without AHD (i.e., 4.1 versus 0.9 per 100 persons-years) and higher among those with moderate and severe acute malnutrition. These results suggest that early screening and diagnosis of AHD and prompt initiation of ART might reduce the number of deaths in this age group.

In Uganda, 30% of newly identified PLHIV (all ages) present for care with CD4 cell counts ≤200cells/mm3 and about 25% return to care with AHD after treatment interruption [12]. The AHD burden of 18% observed in this study show that CALHIV contribute a large share of the country’s AHD burden. The proportion of CALHIV with AHD in this study is similar to those of other studies [14,15] among 15–19 year olds but higher among younger age groups than what has been reported elsewhere [16] and unacceptable in this era of test-and-treat for all PLHIV.

A higher proportion of AHD and mortality were observed among males than females. Evidence elsewhere [17] highlighted greater proportions of undetectable viral loads and survival among females compared to males. A deep dive in our data shows that among those with baseline CD4 count values (1409, males = 500 and females = 909), 103 (21%) of males had baseline CD4 count below 200 copies compared to 138 (15%) among females. These observed differences could point to delays in seeking HIV care among males as seen elsewhere [18,19] and potentially highlight a future research question of whether sex differentials, including potential effects of stigma, in communities influence male CALHIV.

The high proportion (54%) of CALHIV with AHD among those who had been on ART for 12 months or less is not surprising because this is the time when critical assessments, including baseline CD4 count and TB screening, are done. It is also within this critical period that CALHIV are adapting to the new reality of daily swallowing of ART amidst existing HIV-related stigma [20]. However, it is important to note that the proportion with AHD after two years on ART was surprising, because we would expect most of these clients to have stabilised with improved CD4 counts and no stage 3 or 4 AIDS-defining illnesses [21,22]. A high proportion (62.9%, 170/270) with AHD who had been on ART for more than 2 years, being 5–14 years old, calls for a revisit of the psychosocial support, including ART adherence available for this age group both at home and at school.

Our study also highlights that HIV treatment outcomes among CALHIV with AHD are not as good as expected, with approximately 22.1% either dead or LTFU. The finding that deaths among CALHIV with AHD was three times higher compared to those without AHD (8.3% vs 2.8%) signifies the need to prioritize them if we are to achieve set targets under the new global alliance for ending AIDS in children by 2030 [23]. A higher proportion (15.9%) who died in this study among those on ART for 6 months or less was concerning and suggests the need to identify existing health system gaps earlier or provide high quality care after AHD diagnosis. To the best of our knowledge, this study is among the few if not the first to document treatment outcomes specific to CALHIV with AHD. Available published studies [16,24] assessed rates of mortality and LTFU among CALHIV irrespective of their AHD status.

With more than a quarter (26.4%) transferred from their primary HIV care settings and most (74.4%) of these coming from HCIII (36.6%) and hospital (37.8%) levels might point to capacity gaps in managing such cases at those lower levels. Similar gaps have also been identified in the THRIVE project [25] aimed at closing gaps in AHD management among children. The high proportion of patients who TO and/or LTFU has also been observed elsewhere [16] and is often associated with poor documentation of treatment outcomes [26,27] and as such could have underestimated the mortality rate in our study. A robust referral mechanism including health facility-community referral and linkage frameworks would be vital in not only guiding follow-up of clients but also accurately quantifying the burden of AHD in this group.

The risk of mortality of 4.1 deaths per 100 pya among those with AHD compared to 0.9 per 100 pya among those without AHD underscores how vulnerable such clients are and requires more attention as recommended elsewhere [8,28]. In a similar 2010 study in Kenya among children aged between 18 months and 12 years, the mortality rate was 8.4 deaths per 100 child-years [29], which is higher than what we have observed in this study (3.9 per 100 pya [5–9years] and 6.5 per 100 pya [0–4 years]). Higher rates were also seen in another 2014 study among hospitalised children with an overall mortality risk at 6 months follow-up being 61 deaths per 100 pya [30]. These studies could have observed higher mortality rates because children were enrolled from paediatric wards as opposed to this study that used data from out-patient clinics. Additionally, a large part (~59%) of the cohort used in this study had enrolled on more efficacy dolutegravir-based regimens which the country rolled out in 2018. A related 2021 study among PLHIV aged 10 years and older with AHD showed high survival rates (93%) at 12 months, regardless of the presence of opportunistic infections [31] – an indication that risk of mortality has reduced over time but there is more to be done.

A higher risk of mortality among CALHIV with malnutrition (Table 4) aligns with evidence documented elsewhere [32,33] and calls for both client-side and health-system-side interventions in the management of malnutrition. Our study could not elicit if malnutrition is a cause or an effect of AHD, but these findings underscore the increased proportion of deaths if one had both AHD and severe malnutrition. The need to explore the effects of malnutrition on mortality among CALHIV diagnosed with AHD and the leading cause of mortality is henceforth justified.

The increased risk of death particularly among those who had been on ART for less than 6 months highlights the importance of the first year on ART in changing the tide and reducing AHD-related mortalities in this age group. This finding is not surprising as poor immunological response and increase risk of death within the first 6 months after starting ART have also been documented elsewhere [29,34,35]. Existing risk of death (Fig. 1) even after 24 months on ART relates to what has been documented elsewhere [36] and a finding that suggests that not following the national guidelines recommendations to improve ART adherence and continue screening and prophylaxis for opportunistic infections could have negative impacts on the health and lives of CALHIV.

### Study limitations

Our study had some limitations. First, a sizeable proportion (176/382, 46%) of CALHIV were categorised as having AHD based on WHO clinical stage 3 or 4 but with no documented defining illness to qualify the stage 3 or 4. This finding could have overestimated the burden of AHD in this age group. However, since the EMR is a Ministry of Health reference source, we have high confidence in the clinical judgement of providers in these facilities. Secondly, even if the MUAC tape might be limited to children younger than 60 months of age in assessing nutrition status, the 2022 Uganda Ministry of Health guidelines [13] in addition to the weight for age and BMI for age z-scores, also provides for the use of MUAC tapes in patients older than 60 months of age. As such MUAC values used for CALHIV 60 months or older could have resulted in misclassification of the nutrition status in this study.

## Conclusion

Results show overall AHD among CALHIV to be 18.1% and pronounced among those younger than 5 years of age and those on ART ≤ 12 months. CALHIV with AHD and with malnutrition are more likely to die even when on ART. Capacity building of healthcare providers especially at lower-level healthcare facilities to screen and manage AHD as well as provide recommended prophylaxis will be critical. Further research is needed to determine whether malnutrition is a cause or an effect of AHD, as well as the contribution of TO and/or LTFU on the true estimates of AHD and mortality among CALHIV.
